# Competency profiles for evidence-informed policy-making (EIPM): a rapid review

**DOI:** 10.1186/s12961-023-00964-0

**Published:** 2023-02-08

**Authors:** Jorge Otávio Maia Barreto, Davi Mamblona Marques Romão, Cecilia Setti, Maria Lúcia Teixeira Machado, Rachel Riera, Romeu Gomes, Silvio Fernandes da Silva

**Affiliations:** 1 Hospital Sírio-Libanês (HSL), São Paulo, Brazil; 2 Fundação Oswaldo Cruz (Fiocruz), Brasília, Brazil; 3 Instituto Veredas, São Paulo, Brazil; 4 Universidade Federal de São Carlos (UFSCar), São Paulo, Brazil; 5 Universidade Federal de São Paulo (Unifesp), São Paulo, Brazil; 6 Fundação Oswaldo Cruz (IFF/Fiocruz), Rio de Janeiro, Brazil

**Keywords:** Evidence-informed policy-making, Evidence-informed decision-making, Knowledge translation, Competency profiles, Knowledge, Skills and attitudes

## Abstract

**Background:**

Evidence-informed policy-making (EIPM) requires a set of individual and organizational capacities, linked with background factors and needs. The identification of essential knowledge, skills and attitudes for EIPM can support the development of competency profiles and their application in different contexts.

**Purpose:**

To identify elements of competency (knowledge, skills and attitudes) for EIPM, according to different professional profiles (researcher, health professional, decision-maker and citizen).

**Methods:**

Rapid umbrella review. A structured search was conducted and later updated in two comprehensive repositories (BVSalud and PubMed). Review studies with distinctive designs were included, published from 2010 onwards, without language restrictions. Assessment of the methodological quality of the studies was not performed. A meta-aggregative narrative synthesis was used to report the findings.

**Results:**

Ten reviews were included. A total of 37 elements of competency were identified, eight were categorized as knowledge, 19 as skills and 10 as attitudes. These elements were aggregated into four competency profiles: researcher, health professional, decision-maker and citizen. The competency profiles included different sets of EIPM-related knowledge, skills and attitudes.

**Strengths and limitations:**

This study is innovative because it aggregates different profiles of competency from a practical perspective, favouring the application of its results in different contexts to support EIPM. Methodological limitations are related to the shortcuts adopted in this review: complementary searches of the grey literature were not performed, and the study selection and data extraction were not conducted in duplicate.

**Final considerations: conclusions and implications of the findings:**

EIPM requires the development of individual and organizational capacities. This rapid review contributes to the discussion on the institutionalization of EIPM in health systems. The competency profiles presented here can support discussions about the availability of capacity and the need for its development in different contexts.

**Supplementary Information:**

The online version contains supplementary material available at 10.1186/s12961-023-00964-0.

## Background

In the context of health systems, evidence-informed policy-making (EIPM) results from systematic and transparent processes to access, assess, adapt and apply scientific evidence in decision-making processes [1]. EIPM promotes the use of scientific knowledge in decision-making processes and in the development of innovative methods and strategies in the field of health systems. It also fosters technical cooperation between organizations and other interested social groups that produce and apply this scientific knowledge [2].

Thus, EIPM advocates the incorporation of scientific evidence as an input for decision-making processes in the formulation and implementation of health policies. In this context, evidence-informed decision-making (EIDM) emphasizes that decisions should be informed by the best available evidence, as well as other factors such as context, public opinion, equity, feasibility of implementation, accessibility, sustainability and acceptability to stakeholders [3].

In the context of EIDM institutionalization efforts, knowledge translation (KT) is a prior foundation to be considered [3]. Knowledge translation is a dynamic and interactive process that includes synthesis, dissemination, exchange and ethical application of knowledge to improve population health, provide more effective health services and products, and strengthen the health system [4]. This definition is part of a complex system of interactions, also known as knowledge translation platforms [5], which articulates producers, mediators and users of scientific knowledge, in different intensities, complexities and levels of involvement, depending on the nature of the research and the needs in different contexts.

Therefore, four elements of knowledge translation are emphasized: synthesis, dissemination, exchange and practical application of knowledge in the formulation, implementation and evaluation of health policies, at any level of management of health systems and services.

To include scientific evidence in decision-making processes, through systematic, transparent and balanced knowledge translation approaches, it is necessary that individual and institutional capacities are recognized and available. These capacities aim not only to support the use of structured and replicable methods, but also to consider the distinct factors that influence a priority public health problem and the process of implementing interventions to address it. Thus, the decisions to act on the causes and consequences of the problem would be informed in a comprehensive way [6–8].

This set of capacities constitutes a profile, considered from the perspective of professional competencies [9, 10]. The concept of competency considers cognitive, psychomotor and attitudinal attributes as elements of a competent practice [11]. In this regard, competency includes the mobilization of different resources to solve, with relevance and success, problems of professional practice. These resources or attributes are the knowledge, skills and attitudes mobilized, in an integrated way, to conduct professional actions [12, 13].

Although there are studies on the different individual and institutional capacities needed, a global synthesis is not yet available that systematically brings together all these elements, following the logic of competency profiles. Defining the essential competencies for EIPM professionals is key for identifying individual and institutional capacity development needs. This is necessary for establishing knowledge translation platforms in different organizational contexts. In addition, an EIPM competency profile also contributes to the theoretical discussion, but from an applied perspective, supporting the planning and implementation of EIPM initiatives in different contexts.

This study is part of an initiative commissioned by the Brazilian Ministry of Health to support EIPM development in Brazil and aimed to identify EIPM-related competency (knowledge, skills and attitudes). The competency elements were classified according to different professional profiles (researcher, health professional, decision-maker and citizen), considered from a broad conceptual perspective, which can be applied to different socioeconomic contexts and organizational scenarios. The results of this study also supported the development of a specific competency profile for EIPM adapted to the Brazilian context.

## Methods

This study is a rapid umbrella review, which followed a prospective protocol (https://zenodo.org/record/6539137), according to the steps described in this section, including deviations from the protocol. The planning and execution of this review followed the recommendations of the World Health Organization manual for rapid reviews [14] and its report adhered to PRISMA 2020 [15].

### Selection criteria

The following study types were included: overviews of systematic reviews, systematic reviews, scoping reviews and (systematic or narrative) reviews of qualitative studies, that analyzed and/or described professional competencies (knowledge, skills and attitudes) for EIPM, without language restriction, from 2010 onwards (considered by the authors of this rapid review as the time when there has been a growth in global interest in the EIPM institutionalization).

### Review question

The review question was: What are the general and specific competencies (knowledge, skills and attitudes) for professional performance in EIPM? The question was structured according to the population, concept, context (PCC) acronym, as presented in Table 1.

**Table 1:** PCC question (population, concept, context)

Population	Professionals working in EIPM
Concept	Competency profile (skills, knowledge and attitudes)
Context	Any context

### Search strategies and indexed databases

Searches were conducted on two comprehensive and up-to-date databases, BVSalud and PubMed, on 16 March 2022. The search strategies are presented in Table 2.

**Table 2:** Databases and search strategies used

Database	Search strategy
BVSalud	*(knowledge transfer OR knowledge utilization OR knowledge use OR knowledge translation OR knowledge implementation OR research in practice OR knowledge mobilization OR knowledge exchange OR research transfer OR research utilization OR research use OR research dissemination OR knowledge dissemination OR research exchange OR research translation OR knowledge TO action OR know do gap OR evidence informed OR diffusion of knowledge OR research into practice OR knowledge into practice OR evidence into practice OR translational science) AND (competence OR capacity building OR skill OR ability OR training OR curriculum OR learning) AND (type_of_study:(‘policy_brief’ OR ‘sysrev_observational_studies’ OR ‘systematic_reviews’))*
PubMed	((knowledge transfer[Title/Abstract] OR knowledge utilization[Title/Abstract] OR knowledge use[Title/Abstract] OR knowledge translation[Title/Abstract] OR knowledge implementation[Title/Abstract] OR research in practice[Title/Abstract] OR knowledge mobilization[Title/Abstract] OR knowledge exchange[Title/Abstract] OR research transfer[Title/Abstract] OR research utilization[Title/Abstract] OR research use[Title/Abstract] OR research dissemination[Title/Abstract] OR knowledge dissemination[Title/Abstract] OR research exchange[Title/Abstract] OR research translation[Title/Abstract] OR knowledge to action[Title/Abstract] OR know do gap[Title/Abstract] OR evidence informed[Title/Abstract] OR diffusion of knowledge[Title/Abstract] OR research into practice[Title/Abstract] OR knowledge into practice[Title/Abstract] OR evidence into practice[Title/Abstract] OR translational science[Title/Abstract]) AND (competence*[Title/Abstract] OR capacity building[Title/Abstract] OR skill[Title/Abstract] OR ability[Title/Abstract] OR training[Title/Abstract] OR curriculum[Title/Abstract] OR learning[Title/Abstract]))

The protocol of this review included hand searching reference lists of the selected studies and relevant institutional websites. However, we did not consider this necessary to perform because the retrieved studies provided sufficient information for the purpose of this rapid review.

### Screening and selection of studies

Duplicates were excluded, and three reviewers (JOMB, DMMR, CS) independently screened titles, abstracts and full texts, but not in duplicate, supported by the Rayyan platform [16]. Individual doubts were resolved by consensus with a second reviewer (JOMB). Prior to data extraction, a reviewer (CS) read the full texts of selected studies to confirm eligibility.

### Data extraction

One reviewer (CS) extracted data and two other reviewers (JOMB and DMMR) verified the extraction. An electronic spreadsheet was used to systematize the following data from the individual studies selected for inclusion: author, year of publication, purpose of the study, study design, country where the study was carried out, context, target population, competencies identified, barriers and facilitators (when mentioned), knowledge gaps identified by the study, study limitations, conflict of interests declared and funding (when available).

### Data synthesis

We performed a meta-aggregative narrative synthesis [14], based on quantitative and qualitative data from included studies, to combine the individual findings. Two classifications were used to categorize the findings. The first, regarding the competency element, considered the following categories, usually applied in the definition of competency profiles, as the knowledge, skills and attitudes (KSA) model: (1) knowledge: different types of knowledge and information; (2) skills: improved movements and non-verbal communication intertwined with knowledge, expressed as the psychomotor domain in the manipulation and construction of processes and products; (3) attitudes: feelings, positioning and values linked to skills and knowledge in the performance of professional tasks [17]. The second classification considered four professional profiles of interest: (1) researcher: professional who works in the production of scientific research; (2) health professional: professional who works in the provision of health services; (3) health systems and services decision-maker: professional who works in the management of health services and/or systems, at any level; and (4) citizen: individual inserted in civil society, participating or not in organizations representing specific groups.

These categories were used to aggregate the different competency elements identified in this review. This process often led to overlapping elements in the different professional profiles, for example, the same element may be present in more than one profile.

### Methodological quality assessment

We did not perform a methodological quality assessment of the included studies. Although it was included in the protocol of this review, we decided not to proceed with this step because the nature of the question of interest and the scope of this review, and because it would make little contribution to our practical goal.

### Shortcuts adopted and deviations from the protocol

We adopted methodological shortcuts to reduce the time to conduct this rapid review, considering that its purpose was to inform institutional deliberations on a pre-defined schedule. Among the adopted shortcuts, those that potentially influence the completeness and reliability of the findings were: (1) the searches were only performed in the two repositories, including studies published from 2010 onwards, that is, we did not search the grey literature nor the reference list of included studies. This also is a deviation from the protocol, which included complementary searches. Restricting the grey literature search is a common shortcut for rapid reviews for policy topics, as well as tailoring (generally to adjust) the selection of literature databases to the topic, because the addition of a grey literature search depends on the topic, purpose and timeline [14]. In this review, we considered the potential contribution to the topic addressed and the time required for the complementary search, and decided not to extend the searches for grey literature; (2) selection and data extraction were not duplicated but performed individually and verified by another reviewer; (3) the assessment of the methodological quality of the selected studies was not conducted, and this was the second deviation from the protocol. While an assessment of the methodological quality of included studies is desirable in a review, scoping reviews do not require this step, given the potential variety of methodological designs and the nature of the topic or issue addressed [14]; and (4) the results were synthesized with a meta-aggregative approach and presented only descriptively in synthetic tables.

Although these shortcuts and deviations from the protocol suggest caution in the interpretation of the results of this review, they are recognized as potential opportunities to reduce the time spent for the development of rapid reviews that are still reliable [14, 18, 19].

## Results

### Study selection

**Fig. 1:**
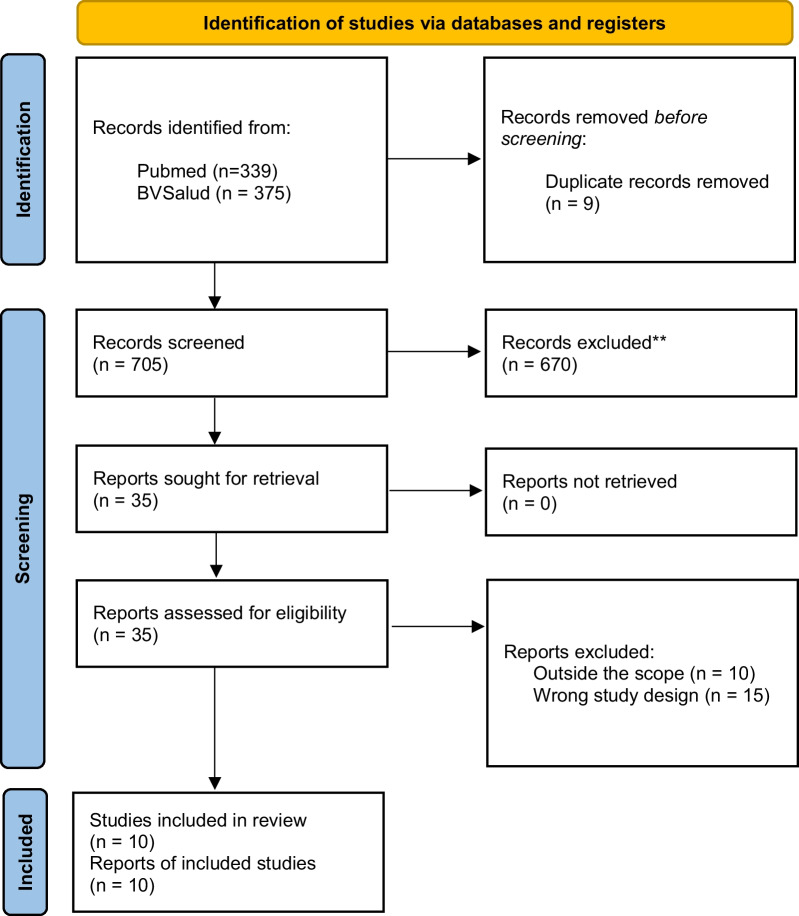
PRISMA flowchart [15]

The searches retrieved 714 documents. Nine duplicates were removed, 705 titles and abstracts were screened, and 35 documents were eligible for full-text reading, 25 of which were excluded for not meeting the inclusion criteria, and two were excluded after data extraction, by consensus of the authors on their eligibility. The list of excluded studies with the reasons for exclusion is provided in Additional file 1: Appendix 1. Ten studies were included in this rapid review (Fig. 1).

### Studies’ characteristics

Among the ten studies included, seven were systematic reviews [7, 20–25], one scoping review [6], one rapid review [26] and one evidence map [8]. The countries of the studies were South Africa [8], Australia [7, 22, 25, 26], Canada [6], United States [24], the Netherlands [21], Iran [20], Norway and Spain [23]. Regarding the target audience, health professionals [6, 21, 22, 24–26], researchers [7], policy-makers [7, 8], managers [6, 20] and citizens [23] were found. Finally, about the researched context, health systems [6, 8, 25], healthcare services [6, 8, 20, 21, 23] and health education sites [7] were included.

### Synthesis of findings

#### General elements of competency in EIPM

Most of the studies included in this rapid review did not explicitly present a framework of ideal competencies for EIPM professionals. However, all included studies reported, according to their purposes, elements that were interpreted to find competencies in EIPM. Thus, the allocation of competencies in the categories adopted (knowledge, skills and attitudes) was made observing the best suitability, according to the authors’ understanding and consensus, as presented in Table 3 and detailed in Additional file 2: Appendix 2.

**Table 3:** Main characteristics of the included studies in this review

Study	Year	Design	Aims	Population	Context	Main findings
Albarqouni L et al. [22]	2018	Systematic review	To assess EBP in educational interventions, review domains of outcomes measured in EBP educational interventions, and assess the psychometric properties of instruments used in studies that assess EBP educational interventions	Health professionals	Not identified in the study	Only 12% of the studies taught content addressed all five EBP steps: (1) ask a question, (2) find information/evidence to answer question, (3) critically appraise the information/evidence, (4) integrate appraised evidence with own clinical expertise and patient’s preferences and (5) evaluate. Sixty-one per cent evaluated EBP skills, 46% knowledge and 41% attitudes
Edwards A et al. [8]	2019	Evidence map	To provide a systematic overview of the literature on knowledge translation (KT) strategies employed by health system researchers and policy-makers in African countries	Health researchers and public health policy-makers	Health systems and services	Commonly reported KT strategies include policy briefs, capacity-building workshops and policy dialogues. Barriers affecting researchers and policy-makers include insufficient skills and capacity to conduct KT activities, time constraints and a lack of resources
Kakemam E et al. [20]	2020	Systematic review	To synthesize the evidence related to leadership and management competencies in health organizations through the best-fit method	Health systems management professionals	Hospitals and community health services	A competency map including seven essential leadership and management competencies
Mallidou AA et al. [6]	2018	Scoping review	To summarize existing knowledge about the (professional) competences needed to implement KT in the health sector	KT professional	Health systems and service	19 essential competences in knowledge, skills or attitudes
Matus J et al. [25]	2018	Systematic review	To identify, evaluate and synthesize existing models and frameworks that describe integrated and practical approaches to building research capacity for allied health professionals in public secondary or tertiary health organizations	Allied health professionals	Secondary/tertiary health organization	Three interconnected and interdependent themes are essential for research capacity building, including ‘supporting clinicians in research’, ‘working together’ and ‘valuing research for excellence’
Oxman AD et al. [23]	2020	Systematic review	To compare the framework provided by the key concepts of Informed Health Choices (IHC) with other frameworks designed to promote critical thinking about claims and treatment (intervention) choices	Citizens, teachers, journalists, researchers and other mediators	Clinical assistance	The IHC framework presents unique competency elements in four distinct categories: (1) recognize when a claim has an untrustworthy basis, (2) recognize when evidence used to support a treatment claim is trustworthy or untrustworthy, (3) make well-informed decisions about treatments and (4) reflect on people’s competences and dispositions
Slade SC et al. [26]	2018	Rapid review	To identify existing research culture frameworks/models and research capacity building and to synthesize existing evidence to identify the essential elements for embedding a research culture within the associated health practice	Health professionals	Not identified	The themes identified as competence elements were: Know how to apply research results to clinical practice. Leadership, mastering research skills and literacy (handling words). Motivation, self-confidence and perceptions of self-worth
Tait H et al. [7]	2019	Systematic review	To describe KT partnership training to health researchers. To examine the assessment approaches used to establish the effectiveness of training in gaining knowledge and skills in KT	Researchers	Teaching in KT	All training programmes included covered practical skills needed for KT, including KT planning and assessment, relationship building and communication, and teamwork
Thompson MR et al. [24]	2019	Systematic review	To discuss the importance and nature of the role of the nurse scientist as a knowledge broke	Nursing researchers	Not identified in the study	Five competencies are central to knowledge brokers: (1) establish (to identify stakeholders), (2) engage (to recognize stakeholders), (3) educate (to facilitate multidirectional knowledge exchanges among the producers and users), (4) empower (to build capacity among stakeholders) and (5) evaluate (to identify resources, processes, outcomes and impacts)
van Dijk N et al. [21]	2010	Systematic review	To evaluate and summarize the literature on the barriers that medical residents experience in applying EBM in daily practice	Health professionals	Health services	Attitudes comprise personal initiative, motivation and interest. Knowledge and skills include clinical questions and evidence-seeking skills, in addition to formal education, critical assessment skills and basic computer skills

KT: knowledge translation; EBP: evidence-based practice; EBM: evidence-based medicine

Competencies were also coded and aggregated, whenever possible, to provide a summarized description of each identified element. The description resulting from this categorization and synthesis process is presented in Table 4, based on the findings of the included studies.

**Table 4:** General list and description of the competency elements (knowledge, skills and attitudes) identified

Knowledge	Description
Knowing the health system context	Knowing the structure and dynamics of the health system, the role of institutions, workers, managers and users [6, 20]
Knowing the organizational context	Knowing the structure and dynamics of the organization(s) in which the policy will be implemented [20]
Knowing basic aspects of health policies	Knowing the basic aspects of health policy formulation, including what they are, how they are designed and how they are implemented [7]
Knowing the fundamentals of academic research	Knowing the basic processes of academic research production, including knowledge of research development tools, research data sources, how to set research priorities and how to conduct research [6, 7, 21]
Knowing group facilitation techniques	Knowing techniques to facilitate group processes, exchange of information, collective construction of knowledge and health practices [6]
Knowing communication techniques	Knowing communication techniques in the context of the health system [6]
Knowing KT methods	Knowing basic processes and methods in KT [6, 7]
Having prior formal education	Having completed higher education and having prior knowledge of foreign languages [6, 21]

Skills	Description
Gaining proficiency in research skills	Gaining proficiency in research skills, knowing how to produce, search, critically assess and synthesize evidence [6–8, 20–23, 26]
Gaining proficiency in management of KT activities	Gaining proficiency in skills related to planning, executing and applying KT strategies [6, 7, 20]
Knowing how to pose relevant questions	Knowing how to identify and prioritize questions relevant to the context of health policies and systems [7, 22]
Knowing how to contextualize evidence	Making use of evidence considering the context of implementation and making the necessary adaptations [6, 7]
Knowing how to apply evidence	Gaining proficiency in ways of applying appropriate evidence in decision-making processes. Knowing how to apply them in accordance with legal practices, recognizing the risks, benefits, biases, effects and costs, maintaining rigor and transparency, and considering the priorities listed [6–8, 20, 22–24, 26]
Knowing how to support the use of evidence by institutions and their key actors	Gaining proficiency in KT strategies to: (a) facilitate the flow of knowledge; (b) improve practice and policy; (c) create demand for evidence; (d) build the policy-maker’s confidence; (d) offer technical support to the needs under discussion; (e) build capacity among stakeholders for evidence-based participatory decision-making; (f) build consensus and support negotiations; (g) assist stakeholders in applying, analyzing and evaluating knowledge in appropriate contexts [6, 7, 24]
Knowing how to communicate evidence to relevant target audiences	Being able to communicate and disseminate the knowledge produced, to promote its use by relevant actors [6, 7]
Knowing how to manage organizations	Knowing how to manage institutions of the public health system [6]
Knowing how to manage people	Knowing how to coordinate teams to achieve institutional goals [6]
Knowing how to manage networks and engage stakeholders	Fostering, developing and nurturing networks between stakeholders, to collaborate in the production and exchange of knowledge (including transdisciplinary), respecting cultural norms and practices, cultivating beneficial and synergistic long-term partnerships whenever possible [7, 24]
Knowing how to manage projects in the public sector	Knowing how to manage resources, processes, risks, and monitor and evaluate projects in the public sector [6, 7, 20, 24]
Knowing how to design public policies	Having the ability to plan and develop programmes and policies for the public sector [24]
Knowing how to implement public policies	Facilitating the implementation of programmes and policies, promoting innovation and the improvement of health practices [6, 7, 24]
Knowing how to do advocacy	Having rhetorical, argumentative or material capacity or potential to negotiate, sustain, defend or propose a certain cause or project with civil society, research groups or institutionally [7]
Knowing how to evaluate public policies	Knowing how to evaluate institutional decisions, processes and results of the policies adopted [7, 20, 22, 24]
Knowing how to establish good interpersonal relationships	Knowing how to establish good interpersonal relationships through: (a) ethical and respectful practices, based on non-violent communication; (b) self-control, self-knowledge, balance and emotional self-management; (c) ability to report and understand information received respectfully, regardless of the hierarchical position occupied [6, 20]
Knowing how to promote cooperative actions	Knowing how to promote, establish and encourage the creation of bonds, partnerships and effective exchanges through cooperation and teamwork between health policy-makers and researchers [6, 7]
Knowing how to lead processes and projects	Knowing how to lead processes and projects, promoting the engagement of the responsible team and relevant key actors [6, 7, 20, 26]
Having basic computer skills	Having basic computer skills, being able to manage essential software and other valuable information technologies for the practice and development of tasks related to EIPM [6, 21]

Attitudes	Description
Acting with professionalism	Acting with high ethical and professional standards, which include integrity, responsibility towards the community, service orientation, commitment to lifelong learning and improvement [6, 20]
Valuing research	Valuing research as a valuable resource for the elaboration of public policy in all its stages [6, 25]
Valuing learning	Having a lifelong commitment to self-directed learning (having an attitude that values experiential learning and persistence, commitment to developing a learning culture and continuous improvement, using critical thinking) [6]
Reflecting carefully	Carefully, judiciously and sensibly reflecting on problems and dilemmas, with a balanced judgment [6]
Acting with creativity	Adopting a creative attitude, seeking to experiment and combine different forms and resources to solve problems [6]
Acting with confidence in one’s own abilities	Making an assertive use of one's already developed knowledge, skills and attitudes [6, 26]
Trusting the other actors in the system	Acting with confidence in the character, integrity and competency of the other actors involved [6]
Appreciating teamwork	Having practices and behaviours that promote and encourage teamwork [6]
Appreciating the possibility of change	Having a flexible personal and professional attitude, accepting, valuing, enabling and managing the occurrence of situations that bring change [20]
Acting with motivation and initiative	Acting with motivation and initiative, proactively seeking opportunities to, in addition to meeting the demands received, contribute to improving the general mood of the environment [21, 26]

KT: knowledge translation

#### Specific elements of competency in EIPM, per professional profile

From the included studies, competency elements were identified and assigned to each professional profile in EIPM: (1) researcher, (2) health professional, (3) decision-maker and (4) citizen. The following Tables 5, 6, 7, 8 present this classification. The studies did not always explicitly associate the competencies with the different profiles. When this association was not mentioned, we assessed the relevance of the competency for each profile and classified them accordingly, based on our understanding of the EIPM field. In the tables, it is indicated whether the competency elements were assigned to each professional profile by the included studies (‘Assigned by the studies’) or, in a complementary way, according to the interpretation of the authors of this rapid review (‘Assigned by the authors’).

**Table 5:** Elements of competency in EIPM, researcher profile

Knowledge	Skills	Attitudes
Assigned by the studies: Knowing the context of the health system [6, 20] Knowing basic aspects of health policies [7] Knowing the fundamentals of academic research [6, 7, 21] Knowing group facilitation techniques [6] Knowing communication techniques [6] Knowing KT methods [6, 7] Having prior formal education [6] Assigned by the authors: Knowing the organizational context	Assigned by the studies: Gaining proficiency in research skills [6–8] Gaining proficiency in management of KT actions [6, 7] Knowing how to pose relevant questions [7] Knowing how to contextualize evidence [6, 7] Knowing how to apply evidence [6–8] Knowing how to support the use of evidence by institutions and their key actors [6, 7, 24] Knowing how to communicate evidence to relevant target audiences [6, 7] Knowing how to manage networks and engage stakeholders [7, 24] Knowing how to manage projects in the public sector [6, 7, 20, 24] Knowing how to design public policies [24] Knowing how to implement public policies [6, 7, 24] Knowing how to do advocacy [7] Knowing how to assess public policies [7, 24] Knowing how to establish good interpersonal relationships [6] Knowing how to promote cooperative actions [6, 7] Knowing how to lead processes and projects [6, 7] Having basic computer skills [6] Assigned by the authors: Knowing how to manage people	Assigned by the studies: Acting with professionalism [6] Valuing research [6] Valuing learning [6] Reflecting carefully [6] Acting with creativity [6] Acting with confidence in one’s own abilities [6] Trusting the other actors in the system [6] Appreciating teamwork [6] Assigned by the authors: Appreciating the possibility of change Acting with motivation and initiative

KT: knowledge translation

**Table 6:** Elements of competency in EIPM, health professional profile

Knowledge	Skills	Attitudes
Assigned by the studies: Knowing the fundamentals of academic research [21] Having prior formal education [21] Assigned by the authors: Knowing the context of the health system Knowing the organizational context Knowing basic aspects of health policies Knowing group facilitation techniques Knowing communication techniques Knowing KT methods	Assigned by the studies: Gaining proficiency in research skills [21, 22, 26] Knowing how to apply evidence [22, 26] Knowing how to assess public policies [22] Knowing how to lead processes and projects [26] Having basic computer skills [21] Assigned by the authors: Knowing how to implement public policies Knowing how to do advocacy	Assigned by the studies: Valuing research [25] Valuing learning [21] Acting with confidence in one’s own abilities [26] Acting with motivation and initiative [21, 26] Assigned by the authors: Acting with professionalism Reflecting carefully Acting with creativity Trusting the other actors in the system Appreciating teamwork Appreciating the possibility of change

KT: knowledge translation

**Table 7:** Elements of competency in EIPM, decision-maker profile

Knowledge	Skills	Attitudes
Assigned by the studies: Knowing the context of the health system [6, 20] Knowing the organizational context [6] Knowing the fundamentals of academic research [6] Knowing group facilitation techniques [6] Knowing communication techniques [6] Knowing KT methods [6] Having prior formal education [6] Assigned by the authors: Knowing basic aspects of health policies	Assigned by the studies: Gaining proficiency in research skills [6, 8, 20] Gaining proficiency in management of KT actions [6] Knowing how to contextualize evidence [6] Knowing how to apply evidence [6, 8, 20] Knowing how to support the use of evidence by institutions and their key actors [6, 24] Knowing how to communicate evidence to relevant target audiences [6] Knowing how to manage organizations [20] Knowing how to manage people [20] Knowing how to manage networks and engage stakeholders [24] Knowing how to manage projects in the public sector [6, 20, 24] Knowing how to design public policies [24] Knowing how to implement public policies [6, 24] Knowing how to assess public policies (20, 24) Knowing how to establish good interpersonal relationships [6, 20] Knowing how to promote cooperative actions [6] Knowing how to lead processes and projects [7, 26] Having basic computer skills [6] Assigned by the authors: Knowing how to pose relevant questions Knowing how to do advocacy	Assigned by the studies: Acting with professionalism [6, 20] Valuing research [6] Valuing learning [6] Reflecting carefully [6] Acting with creativity [6] Acting with confidence in one’s own abilities [6] Trusting the other actors in the system [6] Appreciating teamwork [6] Appreciating the possibility of change [20] Assigned by the authors: Acting with motivation and initiative

KT: knowledge translation

**Table 8:** Elements of competency in EIPM, citizen profile

Knowledge	Skills	Attitudes
Assigned by the studies: Not found Assigned by the authors: Knowing the context of the health system Knowing basic aspects of health policies Knowing the fundamentals of academic research Knowing group facilitation techniques Knowing communication techniques Knowing KT methods	Assigned by the studies: Gaining proficiency in research skills [23] Knowing how to apply evidence [23] Assigned by the authors: Knowing how to do advocacy Knowing how to establish good interpersonal relationships Knowing how to promote cooperative actions	Assigned by the studies: Not found Assigned by the authors: Acting with professionalism Valuing research Valuing learning Reflecting carefully Acting with creativity Acting with confidence in one’s own abilities Trusting the other actors in the system Appreciating teamwork Appreciating the possibility of change Acting with motivation and initiative

KT: knowledge translation

## Discussion

This rapid review addressed a topic of high relevance for EIPM at a global level. The adoption of competency profiles is a critical strategy to support the institutionalization of scientific evidence as an input for decision-making in the formulation and implementation of health policies, in all contexts. A systematic and transparent process was adopted to identify the relevant elements to develop competency profiles for professionals who work in Knowledge Translation and EIPM.

Some earlier studies included in this comprehensive review presented competencies related to knowledge translation and EIPM, but with approaches limited to specific profiles [7, 20–24]. To our knowledge, this is the first study that aggregates different competency profiles.

The findings of this review showed that there are earlier frameworks of competencies in EIPM that can be incorporated into contextualized discussions, at various levels of health policies and systems. These frameworks present elements of competencies that can be classified as knowledge, skills and attitudes (KSA). These competencies, in turn, must be seen as an integrated and interactive set of individual capacities, which interacts with the organizational environment, to constitute professional profiles with different areas of activity. Despite the profiles being different from each other, the overlapping of some elements was common. Moreover, we acknowledge the need to conduct the reclassification and fill the gaps that a rigid classification may produce on these results.

It is also important to emphasize that the practical application of this competencies profile must be broadly anchored in the local needs of each institution and/or professional. Advancing the institutionalization of EIPM requires the recognition of the capacities already available in an institution, which must be compared with the organization’s tasks and attributions. It is this contextualization process that will generate the proper competency profile for each situation. Therefore, this study should be seen as a first input. Its application requires understanding the relevance of each element described here to each organization. For example, the competency elements presented above do not need to be associated with a single professional but can guide the composition of a team that has the necessary set of skills.

Within the EIPM scope, there is a relevant movement aimed at strengthening the institutionalization of knowledge translation processes within governments, civil society organizations and academic institutions [27–29]. However, the lack of tools and frameworks focused on institutional and individual capacities is still a barrier to be overcome. The results of this review provide an acknowledgement of the global literature related to the individual capacities needed, and information that can be immediately applied in discussions and deliberations on the institutionalization of EIPM, in all parts of the world.

### Strengths and limitations

The strengths of this rapid review include: (1) being the first to cover different professional profiles, and adopting a friendly format in the categorization and presentation of the findings to allow the immediate use of its results; (2) adopting systematic and transparent methods to provide, in a timely manner, a body of evidence on an issue of high interest in the current EIPM field, inside and outside Brazil; and (3) contributing to identifying and filling gaps related to the situational diagnosis of individual and organizational competencies for EIPM.

As previously mentioned, methodological limitations include: (1) being a rapid review, we adopted shortcuts and deviations from the protocol, which may have led to the loss of relevant documents, especially from the grey literature. However, we believe that the set of published studies included in this review has sufficiently provided an overview of the available competency elements; (2) the meta-aggregative synthesis carried out to consolidate the results of the different studies included had a narrative character and may have oversimplified the concepts and definitions presented in the description tables of the competency elements. We believe that the guidance to apply the findings of this review in a manner adapted to each contexts’ needs can minimize this limitation, as it will imply a process of re-signification of the findings; (3) the categories used to classify the competency profiles may not be so distinguishable in practice, including elements that are dynamically and interactively correlated. Knowledge, skills and attitudes should be seen as an integrated set of capacities. In the same way, because often there are overlaps and intersections in the profiles presented here, areas of activity should be recognized, rather than actual professional profiles.

## Conclusions

This rapid umbrella review presented elements for professional competency profiles applied to EIPM, contributing to the discussion on the institutionalization of scientific evidence as inputs to systematic, transparent and balanced processes, within the scope of public health policies. The use of these findings will show their usefulness to support strategic planning in health organizations as well as civil society and academic organizations.

## Supplementary Information

**Additional file 1. Appendix 1. **List of excluded studies, after reading the full text, with causes.

**Additional file 2. Appendix 2. **Features of the included studies.

## Data Availability

Available in Additional file 1: Appendix 1 and Additional file 2: Appendix 2.
