# Supplementary material for: Competency profiles for evidence-informed policy-making (EIPM): a rapid review
Source: Health Res Policy Syst. 2023 Feb 8;21:16. doi: 10.1186/s12961-023-00964-0 (PMC9909856; doi:10.1186/s12961-023-00964-0)
Supplement: Supplementary file 1 — Additional file 1. Appendix 1. List of excluded studies, after reading the full text, with causes. [file 12961_2023_964_MOESM1_ESM.docx]

# Additional file

**Appendix 1.** List of excluded studies, after reading the full text, with causes

| **Study** | **Reason** | **Reference** |
| --- | --- | --- |
| [Anderson et al. 2021](https://doi.org/10.1186/s12909-021-02690-z) | Wrong study design | [Anderson, B.J., Dudla, S., Marantz, P.R. et al. Survey analysis to determine the impact of evidence informed practice](https://doi.org/10.1186/s12909-021-02690-z) [education upon East Asian medicine faculty clinical instruction and students’ skills, knowledge, attitudes and](https://doi.org/10.1186/s12909-021-02690-z) [behaviors within a master’s degree program. BMC Med Educ 21, 256 (2021). https://doi.org/10.1186/s12909-021-02690-z](https://doi.org/10.1186/s12909-021-02690-z) |
| [Armstrong et al. 2013](https://doi.org/10.1186/1748-5908-8-121) | Wrong study design | [Armstrong, R., Waters, E., Dobbins, M. et al. Knowledge translation strategies to improve the use of evidence in public](https://doi.org/10.1186/1748-5908-8-121) [health decision making in local government: intervention design and implementation plan. Implementation Sci 8, 121](https://doi.org/10.1186/1748-5908-8-121) [(2013). https://doi.org/10.1186/1748-5908-8-121](https://doi.org/10.1186/1748-5908-8-121) |
| [Charlene et al., 2012](https://doi.org/10.4037/ccn2012159) | Wrong study design / Outside the scope | [Charlene A. Winters, Rebecca Echeverri; Teaching Strategies to Support Evidence-Based Practice. Crit Care Nurse 1](https://doi.org/10.4037/ccn2012159) [June 2012; 32 (3): 49–54. doi: https://doi.org/10.4037/ccn2012159](https://doi.org/10.4037/ccn2012159) |
| [Christopheret al.,](https://doi.org/10.1007/s13142-017-0486-0) [2017](https://doi.org/10.1007/s13142-017-0486-0) | Wrong study design | [Christopher M. Shea, Tiffany L. Young, Byron J. Powell, Catherine Rohweder, Zoe K. Enga, MPH, Jennifer E. Scott, Lori](https://doi.org/10.1007/s13142-017-0486-0) [Carter-Edwards, Giselle Corbie-Smith. Researcher readiness for participating in community-engaged dissemination](https://doi.org/10.1007/s13142-017-0486-0) [and implementation research: a conceptual framework of core competencies, Translational Behavioral Medicine,](https://doi.org/10.1007/s13142-017-0486-0) [Volume 7, Issue 3, September 2017, Pages 393–404, https://doi.org/10.1007/s13142-017-0486-0.](https://doi.org/10.1007/s13142-017-0486-0) |
| Damari et al., 2017 | Wrong study design | Damari B, Ehsani Chimeh E. Public Health Activist Skills Pyramid: A Model for Implementing Health in All Policies. Soc Work Public Health. 2017;32(7):407-420. doi: 10.1080/19371918.2017.1344600. Epub 2017 Aug 11. PMID: 28799849. |
| [Hanney et al., 2020](https://www.ncbi.nlm.nih.gov/books/NBK558508/) | Outside the scope | [Hanney S, Kanya L, Pokhrel S, et al. What is the evidence on policies, interventions and tools for establishing and/or](https://www.ncbi.nlm.nih.gov/books/NBK558508/) [strengthening national health research systems and their effectiveness? [Internet] Copenhagen: WHO Regional Office](https://www.ncbi.nlm.nih.gov/books/NBK558508/) [for Europe; 2020. (Health Evidence Network Synthesis Report, No. 69.) 1. INTRODUCTION. Available from:](https://www.ncbi.nlm.nih.gov/books/NBK558508/) <https://www.ncbi.nlm.nih.gov/books/NBK558508/> |
| Hickman et al., 2018 | Outside the scope | Hickman LD, DiGiacomo M, Phillips J, Rao A, Newton PJ, Jackson D, Ferguson C. Improving evidence based practice in postgraduate nursing programs: A systematic review: Bridging the evidence practice gap (BRIDGE project). Nurse Educ Today. 2018 Apr;63:69-75. doi: 10.1016/j.nedt.2018.01.015. Epub 2018 Jan 31. PMID: 29407264. |
| Hoekstraet al., 2020 | Outside the scope | Hoekstra F, Mrklas KJ, Khan M, McKay RC, Vis-Dunbar M, Sibley KM, Nguyen T, Graham ID; SCI Guiding Principles Consensus Panel, Gainforth HL. A review of reviews on principles, strategies, outcomes and impacts of research partnerships approaches: a first step in synthesising the research partnership literature. Health Res Policy Syst. 2020 May 25;18(1):51. doi: 10.1186/s12961-020-0544-9. PMID: 32450919; PMCID: PMC7249434. |
| Huckel et al., 2014 | Wrong study design | Huckel Schneider C, Campbell D, Milat A, Haynes A, Quinn E. What are the key organisational capabilities that facilitate research use in public health policy? Public Health Res Pract. 2014 Nov 28;25(1):e2511406. doi: 10.17061/phrp2511406. PMID: 25828445. |
| Kahlke et al., 2020 | Outside the scope | Kahlke RM, McConnell MM, Wisener KM, Eva KW. The disconnect between knowing and doing in health professions education and practice. Adv Health Sci Educ Theory Pract. 2020 Mar;25(1):227-240. doi: 10.1007/s10459-019-09886-5. Epub 2019 Mar 23. PMID: 30904958. |
| [Kislov et al., 2014](https://doi.org/10.1186/s13012-014-0166-0) | Wrong study design | [Kislov R, Waterman H, Harvey G, et al. Rethinking capacity building for knowledge mobilisation: developing multilevel](https://doi.org/10.1186/s13012-014-0166-0) [capabilities in healthcare organisations. Implementation Sci 9, 166 (2014). https://doi.org/10.1186/s13012-014-0166-0](https://doi.org/10.1186/s13012-014-0166-0) |
| Leeman et al., 2017 | Outside the scope | Leeman J, Calancie L, Kegler MC, Escoffery CT, Herrmann AK, Thatcher E, Hartman MA, Fernandez ME. Developing Theory to Guide Building Practitioners' Capacity to Implement Evidence-Based Interventions. Health Educ Behav. 2017 Feb;44(1):59-69. doi: 10.1177/1090198115610572. Epub 2016 Jul 10. PMID: 26500080; PMCID: PMC5330318. |
| Leung K et al., 2014 | Outside the scope | Leung K, Trevena L, Waters D. Systematic review of instruments for measuring nurses' knowledge, skills and attitudes for evidence-based practice. J Adv Nurs. 2014 Oct;70(10):2181-95. doi: 10.1111/jan.12454. Epub 2014 May 27. PMID:24866084. |
| Mallidou et al., 2017 | Outside the scope | Mallidou AA, Atherton P, Chan L, Frisch N, Glegg S, Scarrow G. Protocol of a scoping review on knowledge translation competencies. Syst Rev. 2017 May 2;6(1):93. doi: 10.1186/s13643-017-0481-z. PMID: 28464858; PMCID:PMC5414292. |
| [Mihalicza et al., 2018](https://doi.org/10.1186/s12961-018-0331-z) | Outside the scope | [Mihalicza P, Leys M, Borbás I, et al. Qualitative assessment of opportunities and challenges to improve evidence-](https://doi.org/10.1186/s12961-018-0331-z) [informed health policy-making in Hungary – an EVIPNet situation analysis pilot. Health Res Policy Sys 16, 50 (2018).](https://doi.org/10.1186/s12961-018-0331-z) <https://doi.org/10.1186/s12961-018-0331-z> |
| Mishra et al., 2011 | Wrong study design / Outside the scope | Mishra L, Banerjee AT, MacLennan ME, Gorczynski PF, Zinszer KA. Wanted: interdisciplinary, multidisciplinary, and knowledge translation and exchange training for students of public health. Can J Public Health. 2011 Nov-Dec;102(6):424-6. doi: 10.1007/BF03404192. PMID: 22164551; PMCID: PMC6974087. |
| [Padek et al., 2015](https://doi.org/10.1186/s13012-015-0304-3) | Wrong study design / Outside the scope | [Padek, M., Colditz, G., Dobbins, M. et al. Developing educational competencies for dissemination and implementation](https://doi.org/10.1186/s13012-015-0304-3) [research training programs: an exploratory analysis using card sorts. Implementation Sci 10, 114 (2015).](https://doi.org/10.1186/s13012-015-0304-3) [https://doi.org/10.1186/s13012-015-0304-3.](https://doi.org/10.1186/s13012-015-0304-3) |
| Plamondon et al., 2019 | Wrong study design | Plamondon KM, Pemberton J. Blending integrated knowledge translation with global health governance: an approach for advancing action on a wicked problem. Health Res Policy Syst. 2019 Mar 4;17(1):24. doi: 10.1186/s12961-019-0424-3. PMID: 30832660; PMCID: PMC6399857. |
| Roe et al., 2012 | Wrong study design | Roe, Elizabeth Ann PhD, RN; Whyte-Marshall, Mary MSN, OCN, RN-BC Mentoring for Evidence-Based Practice, Journal for Nurses in Staff Development: July/August 2012 - Volume 28 - Issue 4 - p 177-181. doi: 10.1097/NND.0b013e31825dfb2a |
| Scarlett et al., 2020 | Outside the scope | Scarlett J, Forsberg BC, Biermann O, Kuchenmüller T, El-Khatib Z. Indicators to evaluate organisational knowledge brokers: a scoping review. Health Res Policy Syst. 2020 Aug 24;18(1):93. doi: 10.1186/s12961-020-00607-8. PMID: 32831095; PMCID: PMC7444249. |
| [Smits et al., 2018](https://doi.org/10.1186/s12961-018-0393-y) | Wrong study design | [Smits, P., Denis, JL., Préval, J. et al. Getting evidence to travel inside public systems: what organisational brokering](https://doi.org/10.1186/s12961-018-0393-y) [capacities exist for evidence-based policy? Health Res Policy Sys 16, 122 (2018). https://doi.org/10.1186/s12961-018-](https://doi.org/10.1186/s12961-018-0393-y) [0393-y](https://doi.org/10.1186/s12961-018-0393-y) |
| Tabak et al., 2017 | Wrong study design | Tabak, R. G., Padek, M. M., Kerner, J. F., Stange, K. C., Proctor, E. K., Dobbins, M. J., ... & Brownson, R. C. (2017). Dissemination and implementation science training needs: insights from practitioners and researchers. American journal of preventive medicine, 52(3), S322-S329. |
| Uneke et al., 2017 | Wrong study design | Uneke CJ, Sombie I, Keita N, Lokossou V, Johnson E, Ongolo-Zogo P. An assessment of policymakers' engagement initiatives to promote evidence informed health policy making in Nigeria. Pan Afr Med J. 2017 May 24;27:57. doi: 10.11604/pamj.2017.27.57.9844. PMID: 28819479; PMCID: PMC5554684. |
| Appleby et al., 2015 | Outside the scope | Appleby B, Roskell C, Daly W. What are health professionals' intentions toward using research and products of research in clinical practice? A systematic review and narrative synthesis. Nurs Open. 2015 Dec 17;3(3):125-139. doi: 10.1002/nop2.40. PMID: 27708823; PMCID: PMC5047343. |
| Belita et al., 2020 | Outside the scope | Belita E, Squires JE, Yost J, Ganann R, Burnett T, Dobbins M. Measures of evidence-informed decision-making competence attributes: a psychometric systematic review. BMC Nurs. 2020 May 27;19:44. doi: 10.1186/s12912-020-00436-8. PMID: 32514242; PMCID: PMC7254762. |
