# Supplementary material for: Competency profiles for evidence-informed policy-making (EIPM): a rapid review
Source: Health Res Policy Syst. 2023 Feb 8;21:16. doi: 10.1186/s12961-023-00964-0 (PMC9909856; doi:10.1186/s12961-023-00964-0)
Supplement: Supplementary file 2 — Additional file 2. Appendix 2. Features of the included studies. [file 12961_2023_964_MOESM2_ESM.docx]

# Additional file

**Appendix 2.** Features of the included studies

| **Study identification** | **Design, Target population, Context and Purposes** | **Elements of competence** | | | | **Barriers and facilitators** | **Strategies to subsidize competence** |
| --- | --- | --- | --- | --- | --- | --- | --- |
|  |  | **Knowledge** | **Skills** | | **Attitudes** |  |  |
| Mallidou AA et al., 2018.  **Authors' country of affiliation:** Canada. | **Design:** Scope review.  **Target population:** KT professional in the healthcare industry.  **Context:** Health systems and services.  **Purposes:** To summarize existing knowledge about the (professional) competences needed to implement KT in the health sector. | To understand the context (specific organizational context of the practical environment, including system, workers, managers and users).  To understand the research process.  To share knowledge (understanding communication techniques and how language facilitates collaborative activities, knowing the theory and practice of group facilitation).  To be aware of evidence resources (knowing available sources of evidence and knowing how to handle them).  To understand KT / EBP processes.  To understand translation and dissemination activities (knowing how to interpret research results for various audiences and uses).  Knowledge of quality improvement methods and tools, communication strategies and health policies and systems.  In addition to the skills described, the study also found indications of minimum formal education required in gray literature. The most frequently required was a master's degree (17 primary studies), followed by a bachelor's degree (4 primary studies) and a PhD (2 primary studies). The minimum experience required with related KT ranged from none (7 primary studies) to more than seven years of experience (3 primary studies). | Collaboration and teamwork.  Leadership.  Sharing knowledge.  Synthesizing knowledge.  Dissemination of research results.  Use of research results (or use of research).  Fostering innovation (ability to use new tools and strategies to improve practices or policies, address issues, evaluate and build service improvement approaches, and assess the impact of an innovation).  *Knowledge broker* (ability to apply KT strategies to facilitate knowledge flow, improve practice and policy)  KT planning related skills, project management, use of information technology, common sense and discretion / tact / diplomacy and resourcefulness. | | Confidence (a personal factor associated with belief in oneself and one's abilities).  Having confidence (having faith in the character, integrity and truth of others).  Valuing research  Lifelong self-directed commitment to learning (having an attitude that values experiential learning and persistence, a commitment to developing a learning culture and continuous improvement; utilizing an attitude of critical thinking).  Appreciating teamwork  Attitudes such as integrity, commitment to professional work ethic and behavior in interacting with contacts, commitment to high standards of professionalism and interest in the latest developments in communication. | Not identified. | Formal and continuing education and organizational support, such as time to review studies, dialogue between management and staff, creation of a culture of EBP appreciation and active communication. In addition to hands-on training, educational sessions and strategies to improve and expand KT skills and leadership and communication strategies, or funding a knowledge translation champion one day a week. |
| Kakemam E et al., 2020.  **Authors' country of affiliation:** Iran. | **Design:** Systematic review.  **Target population:** Health systems management professionals (hospital leadership and management).  **Context:** Hospitals (public and private) and community health services.  **Purposes:** The purpose of this study was to synthesize the evidence related to leadership and management competencies in health organizations through the best-fit method. | Evidence (Evidence-based decision-making): a) Evidence assessment, b) Evidence application and decision-making, c) Decision assessment.  Knowledge (Proven knowledge of the healthcare and the organization environment): a) Knowledge of the healthcare environment, b) Knowledge of the organization, c) Application of knowledge in legal and quality practices. | | Communication (Interpersonal, communication and relationship management qualities): a) Relationship between management and work team, b) Interpersonal communication, c) Personal qualities in communication (self-control, self-awareness, balance, self-management)  Resources (operations, administration and resource management): a) Team management, b) Financial management, c) Organization management.  Leadership (Leading organizations and people): a) Leadership of people and teams, b) Organizational leadership, c) Leadership qualities. | Professionalism (integration of competencies): the ability to align personal and organizational conduct with ethical and professional standards that include responsibility towards the patient and the community, service orientation and commitment to lifelong learning and improvement.  Change (Enabling and Managing Change): a) Change preparation, b) Change implementation and evaluation, c) Leader quality during change. | Not identified. | Not identified. |
| Tait H et al., 2019.  **Authors' country of affiliation:** Australia. | **Design:** Systematic review.  **Target population:** researchers (from healthcare, healthcare management, policymakers and graduate students interested in KT research).  **Context:** Teaching in KT.  **Purposes:** The purposes of this study are twofold. First, to describe any KT and/or IKT partnership training addressed to health researchers with regard to delivery methods, course content and training topics. Second, to examine the assessment approaches used to establish the effectiveness of training in gaining knowledge and skills in KT. | Clinical and assessment skills (application of KT frameworks in practice, assessment of evidence, skills to facilitate practice change and assessment); assess the impact of KT and research; proficiency in the main research translation topics; knowing health policy and research systems; building evidence-based policy environments; knowledge for the formulation of evidence-based health policies; (acquisition, evaluation, adaptation and application of evidence in the formulation of health policies); research expertise for the development of projects in different themes; understand the evidence needs; knowing about: Introduction to health policy and health systems; Introduction to KT (IKT and *End-of-Grant* KT); Definition of research priorities; Conducting research on policies and practices; Measurements of KT models; Research evidence on health policy formulation and health policy implementation; Advocacy on health policies, creation of demands, consensus building and negotiations; KT tools and strategies for stakeholder and end-user engagement; Policy formulation and implementation process; understanding concepts, methods and theories relevant to KT research; investigating the contribution of different disciplinary and methodological approaches to the practice of KT. | | Developing relationships for engagement and exchange with decision makers; communicating research; building policymaker confidence; leadership skills and management of political interference. | Creating effective bonds, partnerships and exchanges between health policymakers and researchers; playing an expert advisory role and providing scientific evidence to guide policy issues; improving capacity for policymakers; planning and execution of KT and KT research; exploring participation and/or engagement with different stakeholder groups. | Facilitators: Overall, all studies reported that their approach to KT training was well-received and (when measured) that the program improved participants' confidence in key KT-related skills. | All training initiatives that used a traditional course structure (workshop of one or more days) included content on the theoretical basis of KT. In addition to teaching researchers about KT theory, many of the programs included in this review also provided information on health policymaking processes to increase participants' knowledge of context aspects of KT work. The inclusion of theoretical aspects of KT and skills-based practical aspects of KT in all training programs indicates a shared view that participants need to be exposed to a wide range of competencies to effectively engage in KT. |
| van Dijk N et al., 2010.  **Authors' country of affiliation:** Netherlands. | **Design:** Systematic review.  **Target population:** Health professional (medical residents).  **Context:** Health services.  **Purposes:** The aim of this study was to systematically evaluate and summarize the literature on the barriers that residents experience in applying EBM in daily practice. | Knowledge in research (where, how and even when to research), basic computer skills and language knowledge. | | Critical appraisal skills. | Priority; responsibility and experience (implying the best use of time to use the EBM), interest, personal initiative and motivation. | Barriers: limited time, attitude, knowledge and skills, and resident-specific barriers (learning environment, barriers based on the team of surgeons [staff disapproval], institutional and health system factors [lack of funding for health care) and inadequate information resources], lack of interest and low motivation to implement EBM, and perception of inadequate resources available. | Potential solutions to overcome important perceived barriers: EBM training, resource pre-assessment and journal clubs; use of handheld devices to overcome barriers in access and time problems and websites specially designed to assist residents in their searches; formal training in English and the translation of articles as solutions to overcome language barriers. |
| Albarqouni L et al., 2018.  **Authors' country of affiliation:** Australia. | **Design:** Systematic review.  **Target population:** Health professionals.  **Context:** Not identified.  **Purposes:** Sistematically assess and cover the five steps of EBP in educational interventions, review domains of outcomes measured in EBP educational interventions, and assess the psychometric properties of instruments used in studies that assess EBP educational interventions. | Among the studies included, the frequency of appearance/mention of the 5 EBP competencies (1: ask; 2: acquire; 3: appraise; 4: apply; 5: assess) in the educational interventions was: acquisition of evidence (n = 52; 3%) assessed EBPA stage-2 skills; asking a clinical question (n = 51; 61%) assessed EBPA stage-1 skills; critical assessment of evidence (n = 30; 36%) assessed EBPA stage-3 skills. Only 10 (12%) studies covered all five stages of EBP. However, the proportion of studies that taught all five steps has increased over time - from 1 study (out of 39; 3%) in the years prior to 2004 to 6 studies (out of 27; 22%) in 2010-2016, with a particular increase in covering stages 4 and 5. | | (61%) assessed skills contained in EBP stage 3 (critical assessment of evidence), which was the most frequently taught stage in EBP educational interventions (n = 63; 74%).  Similar to previous studies [7, 8] we found that most EBP educational interventions evaluated focus on critically evaluating evidence (EBP Step 3), often to the exclusion of other steps (i.e. apply and reflect). If PBE educational interventions remain primarily focused on teaching how to locate and assess evidence, research evidence can be poorly translated into clinical practice. Rather, greater emphasis should be placed on teaching students how to apply it and on evidence in collaboration with individual patients, for example through shared decision-making. | Not identified. | Not identified. | Not identified. |
| Oxman AD et al., 2020.  **Authors' country of affiliation:** Norway and Spain. | **Design:** Systematic review.  **Target population:** Citizens (teachers, journalists, researchers and other mediators).  **Context:** Clinical assistance.  **Purposes:** Comparing the framework provided by the Key Concepts of Informed Health Choices (IHC) with other frameworks designed to promote critical thinking about claims and treatment (intervention) choices. | Recognizing when evidence used to support a treatment claim is reliable or unreliable by: (a) recognizing the assumptions, evidence and reasoning behind treatment claims; b) recognizing unfair treatment comparisons; c) recognizing unreliable summaries of treatment comparisons; d) recognizing when a statistical model and its assumptions are used to support a treatment claim; e) recognizing misleading ways of presenting the effects of a treatment; f) understanding how systematic errors (the risk of bias), random errors (the game of chance) and relevance (applicability) of treatment comparisons may affect the degree of confidence in estimates of treatment effects; g) understanding the extent to which the evidence supports a treatment claim or not).  Reflecting on people's competencies and dispositions: a) monitor how they decide what treatment they claim to believe in and what to do; b) monitoring how people adjust the processes they use to decide what to believe and do to suit the relevance, importance, and nature of different types of claims and treatment choices; c) being aware of when people are making treatment claims). | | Recognizing when a complaint has an unreliable basis: (a) recognizing claims about the effects of treatments; b) questioning the basis for treatment claims; c) thinking carefully about treatment claims before believing them; d) recognizing when a treatment claim is relevant and important and deserves consideration). | Making well-informed decisions about treatments: (a) being aware of cognitive biases when making decisions; b) clarifying and understanding the problem, options and objectives when making a decision; c) recognizing when decisions have irreversible consequences; d) judging the relevance of the evidence used to inform decisions about treatments; e) weighing the advantages and disadvantages of treatments, taking into account the size of treatment effects, how important each outcome is, costs and certainty of evidence; f) communicating with others about the advantages and disadvantages of treatments). | Not identified. | Not identified. |
| Thompson MR et al., 2019.  **Authors' country of affiliation:** United States. | **Design:** Systematic review  **Target population:** Nursing professional researchers / *knowledge broker*  **Context:** Not identified.  **Purposes:** Tracing the emergence and characteristics of the *knowledge broker* role in all disciplines, internationally and in the United States. This article presents the importance and nature of the nurse scientist's role as a knowledge broker. | Assess: identify resources, processes, results and impacts with schedules at the beginning of the project, with the possibility of monitoring developments and progress.  Enabling: building capacity among stakeholders for evidence-based participatory decision-making; working collaboratively with knowledge producers and users to create new transdisciplinary knowledge whenever possible and appropriate. | | Establishment: identify potential stakeholders (i.e. knowledge producers and users); identify issues of stakeholder concern; Providing technical support to the needs under discussion; ensuring that research is used to inform real decisions and priorities, assessing real problems, developing programs and policies, facilitating their implementation and measuring results; building and maintaining networks between knowledge producers and knowledge users.  Education: assisting parties in the application, analysis and assessment of knowledge in appropriate contexts. Being able to connect science and society, forming partnerships through the construction and establishment of networks, in order to facilitate opportunities between producers and users of knowledge. | Commitment: recognize the cultural norms and practices of stakeholders; establish mutual partnerships Beneficial and synergistic between the parties; nurturing long-term, mutually beneficial synergistic partnerships with stakeholders. | Not identified. | The role of the knowledge broker includes three components: forming and sustaining partnerships; facilitating the application of knowledge; and creating new knowledge. There are five core strategies central to each component of the role (forming and sustaining partnerships; facilitating the application of knowledge; and creating new knowledge): establish, engage, educate, enable and evaluate. |
| Matus J et al., 2018.  **Authors' country of affiliation:** Australia. | **Design:** Systematic review.  **Target population:** Health professionals.  **Context:** Publicly funded secondary and tertiary health organizations  **Purposes:** to identify, evaluate and synthesize existing models and frameworks that describe integrated and practical approaches to building research capacity for allied health professionals in public secondary or tertiary health organizations. | Supporting physicians in research (education and training; Opportunities to get involved; Friendly research workplace; Mentorship/guidance; Access to resources; Time and funding protected; Reward and recognition; Support to undertake postgraduate studies, including higher degrees of research; Teams' skill sets). | | Working together (Collaborations and partnerships with other teams, services and organizations; Shared purpose and drivers; Coordinated approach, including team research projects; Shared experience); Valuing research for excellence (visible support for research; Research as a core business; Prioritization of research that is “close to”/relevant to practice; Integration of local research results back into practice). | Opportunities to get involved; research-friendly workplace; mentoring/guidance; Access to resources; Protected time and funding; Reward and acknowledgement. | Not identified. | Not identified. |
| Slade SC et al., 2018.  **Authors' country of affiliation:** Australia. | **Design:** Rapid review.  **Target population:** Health professionals.  **Context:** Not identified.  **Purposes:** This review should inform the future design of an allied health framework to foster a culture of research in allied health practices. As a first step, the primary objectives of this rapid review are (1) to identify existing research culture frameworks/models and research capacity building, as well as (2) to synthesize existing evidence to identify the essential elements for embedding a research culture within the associated health practice. The secondary objective is to summarize the strengths and limitations of existing frameworks and models. | Know how to apply research results to clinical practice. | | Leadership, mastering research skills and literacy (handling words). | Motivation, self-confidence and perceptions of self-worth. | Facilitators: Frameworks for embedding a culture of research in allied health practice: -Overarching theme: Providing research-based health care that is consistent with the best available evidence requires comprehensive policies that allow the organization and individuals to be active in research.  1) Regulatory environment, governance and organizational structures: Sustainable change requires health research policies, regulation, governance and organizational structures that support and value evidence-based practice;  2) Management leadership and adherence: Research capacity, receptivity and literacy of health leaders and managers are critical to the implementation of research;  3) Systems, tools, resources and time: The provision of research infrastructure, research systems, tools, databases, resources, time allocation, dedicated research staff positions, mentoring, professional education, and recognition and reward mechanisms are key organizational factors that enable research empowerment; Partnerships between health agencies and universities with research leaders optimize research quality and productivity;  4) Attributes of individual physicians: Attributes and capabilities of individual physicians, such as research qualifications, skills, literacy in research, communication skills, partnerships, trust and motivation help to strengthen and develop research interactions and increase research receptivity. | Regulation, strong leadership and supportive management structures are essential elements of a successful research culture within allied health. Research skills and research capacity can stabilize and sustain a free research workforce; research evidence is available, accessible and usable by the individual; allocation of time for research and skills acquisition; administrative, technical and information support, including library, computers and software; (providing) measurable outcomes and professional development, for example, PhD, external funding, conference, attendance, publications; research networks, group activities and career paths with access to supervision and mentoring; being available, accessible and usable by the individual; time allocation for research and skills acquisition; administrative, technical and information support, including library, computers and software; (providing) measurable results and professional development, eg. PhD, external funding, conference, attendance, publications; research networks, group activities and career paths with access to supervision and mentoring. |
| Edwards A et al., 2019  **Authors' country of affiliation:** South Africa. | **Design:** Evidence map  **Target population:** Health researchers and public health policymakers involved in non-executive or policy-level management or decision-making about health programs or services.  **Context:** Health systems and services in African countries.  **Purposes:** First, positioning the current available research on KT in health policymaking in Africa within the broader field of KT. Second, providing a summarized and synthesized understanding of the current situation and key issues within a massive and diverse body of literature for researchers and policymakers in the African health system. Finally, generating a user-friendly evidence map, testing the usefulness of the evidence mapping approach for this type of diverse and dispersed topic and for the field of health and research policy of systems more broadly. | Availability of quality research relevant to the local context; leadership and political will for kt; presence of technical support and expertise; regular clear communication using a common language; organizational culture that favors the use of research; access to academic journals and guidelines; clear and comprehensive recommendations; previous experience of policymakers with research; mentoring; framing evidence as a policy issue. | | Ability to generate, understand and use surveys; strong institutional and personal links/networks; partnerships and collaboration; use of knowledge brokers/local champions; formalized organizational processes / structures for kt; trust between researchers and policymakers; Opportunities for mutual learning and building networks; wide range of engaged stakeholders; alignment of research with local priorities; financial resources. | Early and sustained stakeholder engagement over time; credibility (of results, research, organization and network); target communication plan, using various methods; policymakers place a high value on research results; search time; access to the policymaker; researcher characteristics (post-test results, clear policy benefits); understanding of the local context; continuous monitoring and prompt reaction to emerging problems. | Barriers - For policymakers: access to relevant and reliable research and lack of locally applicable research. For researchers: poor communication with decision makers, attitudes of policymakers towards using research on the need for KT and high turnover of government officials. Barriers that mutually affected researchers and policymakers were insufficient skills and ability to carry out KT activities, time constraints and insufficient resources (funding).  Facilitators - Policymakers: the availability of quality research relevant to local contexts, political will for KT and the presence of experienced technical support. Researchers: stakeholder support for the research results was important in facilitating the researcher's efforts in KT. Trust among researchers and policymakers was mentioned as either a facilitator or a barrier. Mutual facilitators are the ability to generate, understand and utilize research results, demonstrating that the presence of KT skills is potentially as important as their absence. Furthermore, strong institutional bonds and networks, partnerships and collaborations, and the use of *knowledge brokers* or local champions highlight the importance of interactions between researchers and policymakers when seeking research to influence policies. | Not identified. |

Notes: EBP: evidence-based practice; EBM: evidence-based medicine; KT: knowledge translation
